# Quantitative mitochondrial DNA copy number determination using droplet digital PCR with single-cell resolution

**DOI:** 10.1101/gr.250480.119

**Published:** 2019-11

**Authors:** Ryan O'Hara, Enzo Tedone, Andrew Ludlow, Ejun Huang, Beatrice Arosio, Daniela Mari, Jerry W. Shay

**Affiliations:** 1 Department of Cell Biology, UT Southwestern Medical Center, Dallas, Texas 75390, USA;; 2 Geriatric Unit, Department of Medical Sciences and Community Health, University of Milan, 20122 Milan, Italy;; 3 Fondazione Ca’ Granda, IRCCS Ospedale Maggiore Policlinico, 20122 Milan, Italy

## Abstract

Mitochondria are involved in a number of diverse cellular functions, including energy production, metabolic regulation, apoptosis, calcium homeostasis, cell proliferation, and motility, as well as free radical generation. Mitochondrial DNA (mtDNA) is present at hundreds to thousands of copies per cell in a tissue-specific manner. mtDNA copy number also varies during aging and disease progression and therefore might be considered as a biomarker that mirrors alterations within the human body. Here, we present a new quantitative, highly sensitive droplet digital PCR (ddPCR) method, droplet digital mitochondrial DNA measurement (ddMDM), to measure mtDNA copy number not only from cell populations but also from single cells. Our developed assay can generate data in as little as 3 h, is optimized for 96-well plates, and also allows the direct use of cell lysates without the need for DNA purification or nuclear reference genes. We show that ddMDM is able to detect differences between samples whose mtDNA copy number was close enough as to be indistinguishable by other commonly used mtDNA quantitation methods. By utilizing ddMDM, we show quantitative changes in mtDNA content per cell across a wide variety of physiological contexts including cancer progression, cell cycle progression, human T cell activation, and human aging.

Human mitochondrial DNA (mtDNA) is a circular 16.6-kb genome residing within the mitochondrial matrix that encodes for 13 protein components of the electron transport chain essential for oxidative phosphorylation (OXPHOS). mtDNA is present at hundreds to thousands of copies per cell, varying widely between cell types and tissues (Robin and Wong 1988; Pierce et al. 1990; Shay et al. 1990). Cellular coordination of mtDNA content is a dynamic and tightly regulated process (Li et al. 2005; Scarpulla 2006; Wu et al. 2006); however, the mechanisms by which mtDNA copy number is monitored and controlled are not well understood (Moraes 2001; Clay Montier et al. 2009; Klingbeil and Shapiro 2009). In addition, alterations in mtDNA levels often accompany key pathophysiological changes during the transition from healthy to diseased states (Butow and Avadhani 2004), and a number of age-related diseases correlate with mtDNA abundance, including cardiovascular disease (Yue et al. 2018), type 2 diabetes (Malik et al. 2009; Monickaraj et al. 2012), cancer (Afrifa et al. 2018), and dementia (Rice et al. 2014; Pyle et al. 2016). Furthermore, mtDNA levels in peripheral blood mononuclear cells (PBMCs) gradually decrease during aging and are associated with health status among the elderly (Mengel-From et al. 2014; Wachsmuth et al. 2016), suggesting that mtDNA may be a biomarker of biological (not chronological) age and disease exposure (Pieters et al. 2015; Qiu et al. 2015; Tyrka et al. 2015).

The growing relevance of mtDNA as a biomarker highlights the need for a more high-throughput method of mtDNA quantification. The current standard employed in the measurement of mtDNA copy number is quantitative PCR (qPCR). Measurements by qPCR require the use of a reference gene and are often displayed as a ratio of mitochondrial to nuclear DNA; however, qPCR is particularly susceptible to differing PCR efficiencies between target and housekeeping genes, leading to skewing of this ratio (Regier and Frey 2010; Kiselinova et al. 2014). Additionally, the use of a reference gene subjects qPCR results to compounding errors, further reducing the sensitivity of qPCR measurement. Recent work has strengthened the utility and flexibility of droplet digital PCR (ddPCR) technology (Ludlow et al. 2014; Huang et al. 2017). The ddPCR technology uses oil emulsion to partition samples into thousands of droplets, each representing an independent PCR system. Since all template-containing droplets reach plateau during the PCR step, complications arising from PCR inhibitors and differing PCR efficiencies are minimized. The total number of droplets and droplets that fluoresce are counted in a flow cytometry-like fashion to produce a ratio that is then subjected to Poisson distribution, resulting in an absolute quantification of starting template molecules (Hindson et al. 2011; Pinheiro et al. 2012; Robin et al. 2016). Methods to quantify mtDNA copy number by ddPCR using purified genomic DNA have recently been developed (Hindson et al. 2011; Pinheiro et al. 2012; Podlesniy et al. 2013; Wachsmuth et al. 2016). However, the time-consuming nature of DNA purification represents a major rate-limiting step in the quantification of nucleic acids (Van Peer et al. 2012) and thus a major hurdle in developing much needed higher-throughput methods for mtDNA copy number evaluation.

Here, we present a new quantitative, highly sensitive ddPCR method, droplet digital mitochondrial DNA measurement (ddMDM), to measure mtDNA content per cell equivalent directly from cell lysates and in single cells without the need for DNA purification or inclusion of nuclear reference genes.

## Results

### ddMDM workflow and experimental design

**Figure 1.**
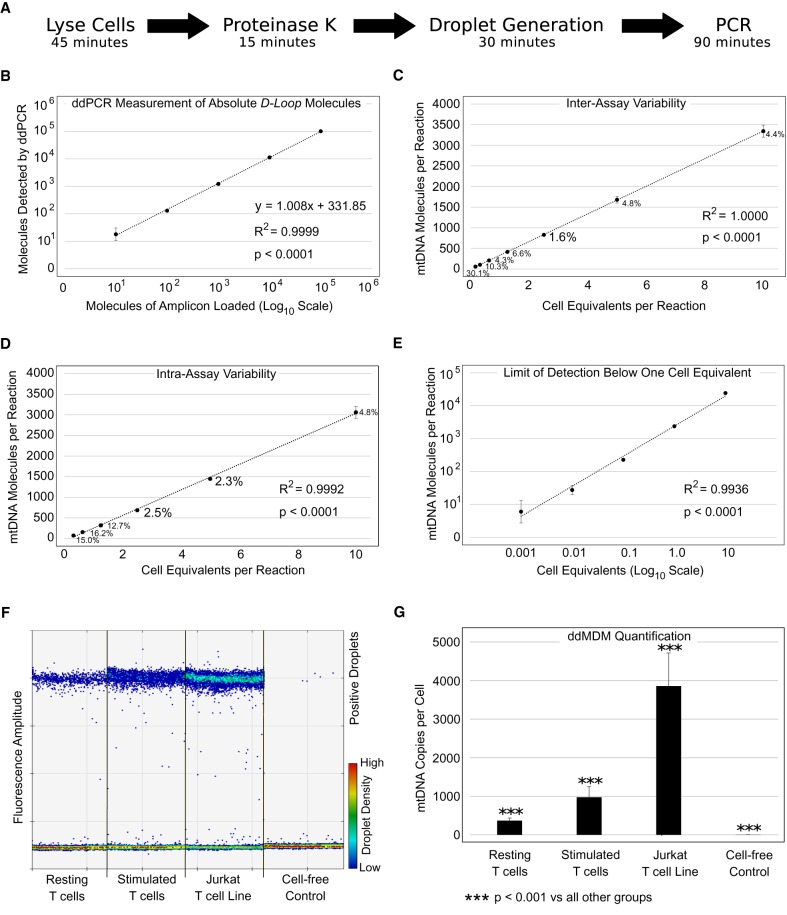
Optimized workflow and validation of the method. (*A*) General workflow of ddMDM for the quantification of mtDNA on a per cell equivalent basis using ddPCR. (*B*) Absolute quantification of *D-Loop* amplicons by ddPCR in a 10-fold dilution series. Axes are displayed in log_10_ scale. Error bars show the standard deviation of four technical replicates. (*C*) Inter-assay variability of ddMDM. Data points represent the mean of means of four biological replicates run in duplicate (seven separate dilution series of early passage BJ fibroblasts’ cell lysates). Adjacent numbers show the coefficient of variation (CV) of the means. (*D*) Intra-assay variability of ddMDM. Data points represent the average of five technical replicates from a single twofold dilution series of early passage BJ fibroblasts. (*E*) Limit of detection. Tenfold dilution series in late passage BJ fibroblasts. Data points show the average of two experiments run in triplicate. Error bars display the standard deviation of the six replicates. (*F*) ddPCR output showing resting and stimulated T lymphocytes, Jurkat T leukemia cells, and a cell-free (lysis buffer) control. Each lane shows a single ddPCR well, containing an input of 5 cell equivalents. Dots represent emulsion droplets. (*G*) Quantification of ddPCR results. mtDNA copy number was calculated per cell equivalent. Error bars show the standard deviation of biological replicates. Significance was determined by ANOVA (Tukey's multiple comparisons).

We optimized the ddMDM workflow for accuracy in quantitation, reproducibility, speed, and higher-throughput applications (Fig. 1A). We initially created three sequences (amplicons) representing the mtDNA *D-Loop*, *MT-ND1*, and *MT-TL1* regions; we made serial dilutions of the amplicons and evaluated the linearity and slopes of three different primer pairs targeting these sequences (Fig. 1B; Supplemental Fig. 1A,B; Supplemental Tables S1, S5). We confirmed that measurements taken by using ddPCR accurately reflected absolute molecules per reaction in a nearly 1-to-1 ratio (Fig. 1B; Supplemental Fig. 1A,B; Supplemental Tables S1, S5). Because large mtDNA deletions encompassing the *D-Loop* region have not been previously reported (Bai and Wong 2005), the primer pair targeting the *D-Loop* region was employed in all subsequent experiments.

Since the results by using serial dilutions of amplicons were highly reproducible (Fig. 1B; Supplemental Table S1), we next evaluated the reproducibility of the assay when cell lysates rather than artificial amplicons are used. To this aim, we evaluated the intra- and the inter-assay variability by measuring the coefficients of variation (CV) across twofold dilution series of BJ fibroblasts cell lysates. We documented very robust reproducibility across seven serial dilutions, with the best reproducibility seen within the range of 800 to 1000 *D-Loop* molecules per reaction (inter-assay CV = 1.6%; intra-assay CV = 2.5%) (Fig. 1C,D; Supplemental Table S1). Consistent with other previously published ddPCR methods (Ludlow et al. 2014), this corresponded to ∼5% of fluorescent (positive) droplets out of the total number of droplets.

We next measured the limit of detection of ddMDM by quantifying mtDNA in a 10-fold dilution series of cell lysates (stimulated T cells) and observed that ddMDM can quantify mtDNA from as little as one hundredth of a cell equivalent (R^2^ = 0.99) (Fig. 1E; Supplemental Table S1). Finally, we tested in a dilution series of cell lysates whether a hydrolysis probe approach (TaqMan) could further improve the accuracy of ddMDM when compared to using EvaGreen, but we observed no difference in mtDNA copy number quantitation between the two fluorophores (Supplemental Fig. 1C; Supplemental Table S5). For this reason, EvaGreen (less expensive and simpler to use than TaqMan probes) was employed in all experiments. Together, these results demonstrate that ddMDM reliably quantitates mtDNA copy number by using cell lysates directly. In addition, we quantitated differences in mtDNA content between resting, stimulated, and transformed T cells (Fig. 1F; Supplemental Table S1). Raw ddPCR output was then transformed into absolute numbers of mtDNA molecules per cell equivalent with a high degree of precision (*P* < 0.001) (Fig. 1G; Supplemental Table S1).

### ddMDM is more sensitive compared to other commonly employed techniques

**Figure 2.**
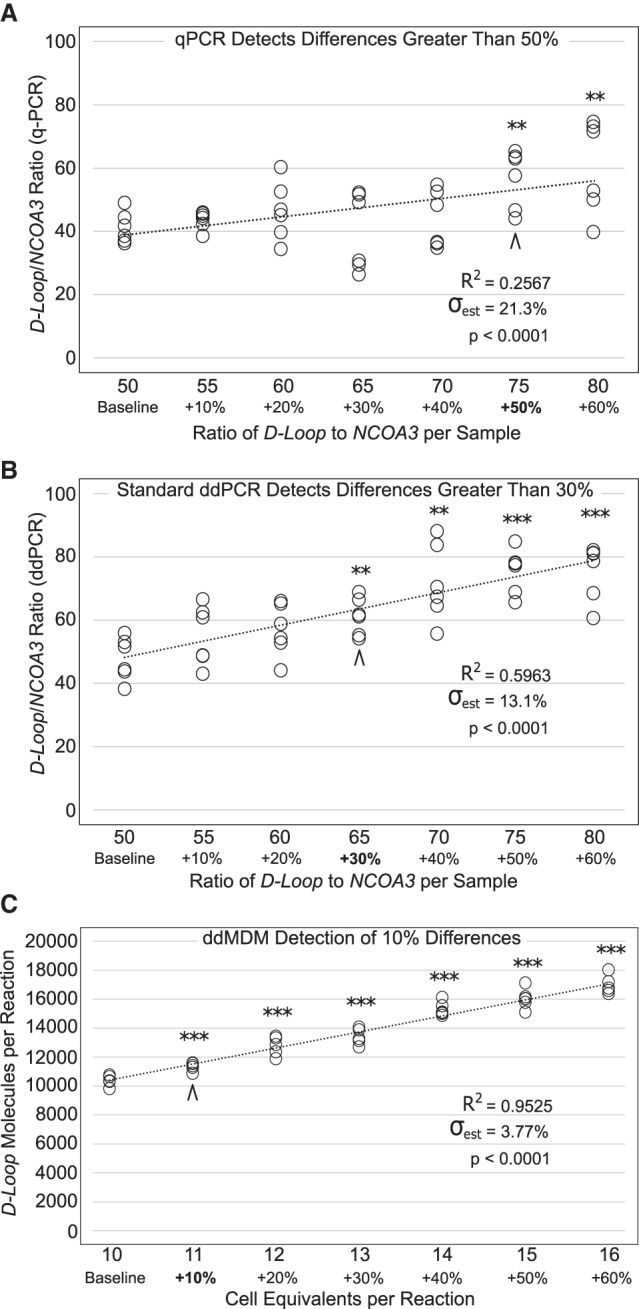
Limit of sensitivity for ddMDM and other previously reported techniques for mtDNA detection. Data points are the result of two separate experiments each performed in triplicate. Asterisks indicate significance by Student's *t*-test between each sample compared to the baseline control. (**) *P* < 0.01, (***) *P* < 0.001. σ_est_ displays the standard error of the estimate as a percentage, normalized to the population mean. (*A*) qPCR determination of *D-Loop*/*NCOA3* ratio using purified amplicons. (*B*) ddPCR determination of *D-Loop*/*NCOA3* ratio of the exact same samples employed in Figure 2A. (*C*) ddMDM determination of mtDNA molecules (*D-Loop*) per cell equivalent (stimulated T cells).

Current techniques for assaying mtDNA copy number are often limited in their ability to detect subtle changes in mtDNA levels. As such, it is unknown at what threshold minor perturbations in cellular mtDNA content begin to impact health and disease. We therefore compared ddMDM's limit of sensitivity to that of other methods by calculating the smallest significant difference that was detectible by each technique. To test this for other techniques, we mixed mitochondrial and genomic amplicons in fixed ratios, simulating samples that differed in mtDNA copy number by set percentages (10% increments in mtDNA) compared to a baseline control. Student's *t*-test was used to compare each sample against the baseline control and one-way ANOVA was employed to compare each sample against every other sample. In addition, the standard error of the estimate (σ_est_) was calculated as a measure of the overall variability in each assay. We observed that qPCR could significantly (*P* < 0.05, Student's *t*-test) distinguish between samples whose mtDNA/nDNA copy number ratio differed by at least 50% (Fig. 2A; Supplemental Table S2), whereas the more stringent ANOVA showed that differences could only be detected at 60% (*P* < 0.05). The σ_est_ for qPCR detection was 21.3%, reflecting how even moderate deviations in threshold cycles (Ct) can produce large changes in relative abundance calculations.

In contrast, ddPCR can detect significant differences (*P* < 0.05, Student's *t*-test) as low as 30% above the baseline, with a σ_est_ of 13.1% (Fig. 2B; Supplemental Table S2). ANOVA showed that all comparisons with >33% difference in sample loaded were significant. The exact same DNA samples were used for both qPCR and ddPCR experiments, emphasizing that ddPCR use of endpoint rather than threshold fluorescence produces less variable results and quantifications closely resembling the sample ratio that was prepared (Pinheiro et al. 2012; Kiselinova et al. 2014; Zhao et al. 2016).

Finally, we tested the limit of sensitivity for ddMDM using a dilution series of cell lysates from stimulated human T lymphocytes. We found that ddMDM was able to distinguish, with a high degree of significance (*P* < 0.001, Student's *t*-test), all samples from the baseline sample (Fig. 2C; Supplemental Table S2). Furthermore, ANOVA showed that samples characterized by at least a 9.1% difference in mtDNA content were significantly different. Unlike standard qPCR and ddPCR assays, ddMDM foregoes the use of a nuclear reference gene and thus eliminates the compounded error caused by variability of an additional sample in calculating copy number. The advantage of this is further highlighted by the σ_est_ of only 3.77% (Fig. 2C; Supplemental Table S2). In summary, we show that ddMDM can detect significant differences between samples whose mtDNA copy number was close enough as to be indistinguishable by other established mtDNA quantitation techniques.

### Detection of mtDNA in a variety of human cells by ddMDM

**Figure 3.**
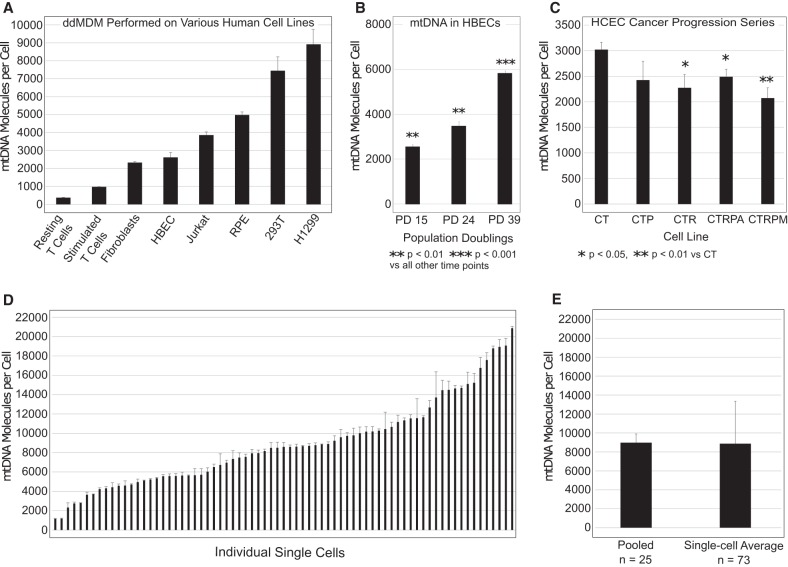
ddMDM quantification of mtDNA in different cell types. (*A*) Absolute mtDNA molecules per cell equivalent were measured in different primary and transformed cell lines. (*B*) mtDNA levels in primary HBECs at the indicated population doublings. (*C*) mtDNA levels in human colonic epithelial cell (HCEC) cancer progression series. Error bars show the standard deviation of biological triplicates. (*D*) mtDNA from 73 single H1299 cells were quantified. Error bars show the standard deviation of three technical replicates for each individual cell. (*E*) Comparison between averages of H1299 pooled controls and of the 73 single cells.

In order to show the utility and flexibility of ddMDM, we evaluated mtDNA content in various cell types and cellular contexts. Initially, we examined differential abundance in mtDNA in normal and transformed human cells, having hundreds to thousands of mtDNA copies per cell (Fig. 3A; Supplemental Table S3). Consistent with previously reported studies, we interpret our results to suggest that mtDNA content varies widely depending on both cell size and cell line-specific metabolic profiles (Robin and Wong 1988; Veltri et al. 1990).

Changes in mtDNA copy number during aging have been shown to be tissue- and cell-type–specific (Wachsmuth et al. 2016). For example, mtDNA levels decrease in human peripheral immune cells during aging (Mengel-From et al. 2014), but they can increase in other cell types. For example, increases in mtDNA content occur during in vitro replicative aging of human bronchial epithelial cells (HBECs) (Fig. 3B; Supplemental Table S3). Consistently, the most significant increase in mtDNA copies per cell occurred at around 3–4 population doublings (PD) prior to replicative senescence (∼PD 42) (Fig. 3B; Supplemental Table S3) and was accompanied by a decreased replicative capacity.

Metabolic reprograming is one of the key hallmarks of cancer (Ward and Thompson 2012), and mtDNA copy number has been reported to correlate with cancer invasiveness and overall patient outcomes (Chen et al. 2016; Hu et al. 2016). In addition, it has been suggested that lower levels of mtDNA in certain cancers may reflect a more stem-like metabolic program and be important in cancer stem cell biology (Lee et al. 2015; Menendez 2015). We used the normal human colonic epithelial cells (HCEC) system with experimentally introduced mutations that commonly occur during colorectal cancer initiation and early progression to examine changes in mtDNA copy number (Eskiocak et al. 2011; Zhang et al. 2015). Specifically, we employed ddMDM to assess how mtDNA abundance varied in response to loss of tumor suppressors (*TP53* and *APC*), oncogenic mutations (*KRAS^v12^),* and *TERT, CDK4*, and *MYC* overexpression in normal HCECs. We found a significant decrease in mtDNA per cell in the context of *KRAS* (1CTR and 1CTRPA) and *KRAS* + *MYC* (1CTRPM) overexpression (*P* < 0.05 and *P* < 0.01 respectively) (Fig. 3C; Supplemental Table S3), suggesting that these cells may be reverting to a more stem-like, undifferentiated state characterized by limited OXPHOS and a preference toward glycolysis (Menendez 2015; Kawada et al. 2017).

### ddMDM for quantitation of mtDNA copy number in single cells

During disease progression, as well as normal aging, key cellular subsets within a mixed cell population often contribute in varying and crucial ways. However, when these peculiar cell subsets are rare, they become diluted and dispersed within the heterogeneous population generally below detection (Mendenhall et al. 2016; Ibrahim-Hashim et al. 2017). In cancer, for example, metabolic reprogramming within tumor subpopulations represents one possible molecular mechanism by which chemotherapy-induced resistance can be acquired (Morandi and Indraccolo 2017). Therefore, in order to further characterize diverse and metabolically distinct T cell subpopulations, efficient methods for measuring mtDNA copy number at the single-cell level are needed.

Therefore, we next investigated whether ddMDM was sensitive enough to reliably measure mtDNA copy number in single cells. Starting with a clonal population, single H1299 human lung cancer cells were plated in 1-µL aliquots onto glass slides and visually confirmed for the presence of exactly one cell by microscopy. Each individual cell was then lysed in a separate tube and 0.3-cell equivalents were employed for the ddMDM assay. Due to the exceptional sensitivity of ddMDM, less than a single-cell equivalent was sufficient to provide an adequate ddPCR signal. This allowed us to perform all single-cell ddMDM in triplicate (from the same individual cell), providing technical replicates for each single-cell reaction. We found that mtDNA content varied widely from cell to cell (Fig. 3D; Supplemental Table S3). However, the average mtDNA content from pooling the results from single cells was nearly identical to that of the pooled mixed population of cells (Fig. 3E; Supplemental Table S3). This suggests that these results reflect extreme intercellular differences in mtDNA copy number rather than some type of technical artifact. Additionally, these large differences between single cells cannot be fully explained by cell cycle-dependent variations in mtDNA copy number (Supplemental Fig. 1D; Supplemental Table S5). Although we observed a significant increase in mtDNA copy number during the transition from G2 to M phase, and a significant decrease during the early G1 phase, these changes were modest when compared to single-cell mtDNA heterogeneity in cancer cells (Fig. 3D; Supplemental Fig. 1D; Supplemental Tables S3, S5). To investigate further whether the observed high intercellular variability in mtDNA content was due to real biological differences between single cells or was due to a limitation in our single-cell isolation method, we seeded H1299 at clonal density and expanded several clones. When clones reached approximately 2–3 × 10^5^ cells (corresponding to ∼18 population doublings), we isolated single cells via FACS and repeated the single-cell mtDNA quantification. We found a consistently lower intercellular variability in mtDNA content in single cells sorted from the same clone (Supplemental Fig. 2; Supplemental Table S6) as compared to single cells from the original heterogeneous population (Fig. 3D; Supplemental Table S3). These data can be interpreted to suggest that the intercellular heterogeneity initially observed can be explained, at least in part, by the presence of varying cell subpopulations in cancer as previously suggested (Januszyk et al. 2015). In summary, these results show that ddMDM can reliably quantify and detect differences in mtDNA content in single cells.

### mtDNA levels during T cell stimulation decrease with age, but healthy centenarians escape this decline

Previously, we identified genes and pathways potentially involved in the process of healthy human immune cell aging and longevity through the study of stimulated PBMCs from a well-characterized population of 114 individuals aged 23–113 yr (Tedone et al. 2019). PBMCs are a heterogeneous cell population mainly consisting of T cells, a major component of human immune responses. T cells remain in a resting or quiescent nonproliferating state when unstimulated, retaining decreased cell size and lower metabolic activity. In contrast, upon antigen-specific activation, T cells rapidly divide and exhibit significant changes in gene expression (Zhao et al. 2014; Tedone et al. 2019) and metabolic features (Dimeloe et al. 2017). Activated T cells initiate immune responses such as discriminating between healthy and abnormal (e.g., infected or cancerous) cells in the body and thus represent a valuable model to study the physiological function of the adaptive immune system. Previous studies have shown a progressive decline of mtDNA copy number in resting PBMCs during aging (Kim et al. 2013; Mengel-From et al. 2014; Wachsmuth et al. 2016). However, comparing stimulated PBMC mtDNA levels in different age groups, including centenarians (100+ yr old), is an understudied area of research and might provide new insights about the mechanisms driving the age-related impairment of the immune system. Thus, we stimulated PBMCs from 22 healthy adults (aged 33 ± 7 yr; hereafter referred to as “young”), 22 elderly adults (aged 73 ± 6 yr; hereafter referred to as “old”), and 17 centenarians (aged 104 ± 4 yr) and employed ddMDM to track changes in mtDNA levels over a 15-d (Young) or 10-d (Old and Centenarian) period following stimulation. Since centenarians’ health status has been reported to strongly correlate with their immune cell replicative potential and gene expression (Tedone et al. 2019), we subdivided our centenarians’ study group into two subgroups: healthier centenarians and frail centenarians (Supplemental Table S7; Tedone et al. 2019).

**Figure 4.**
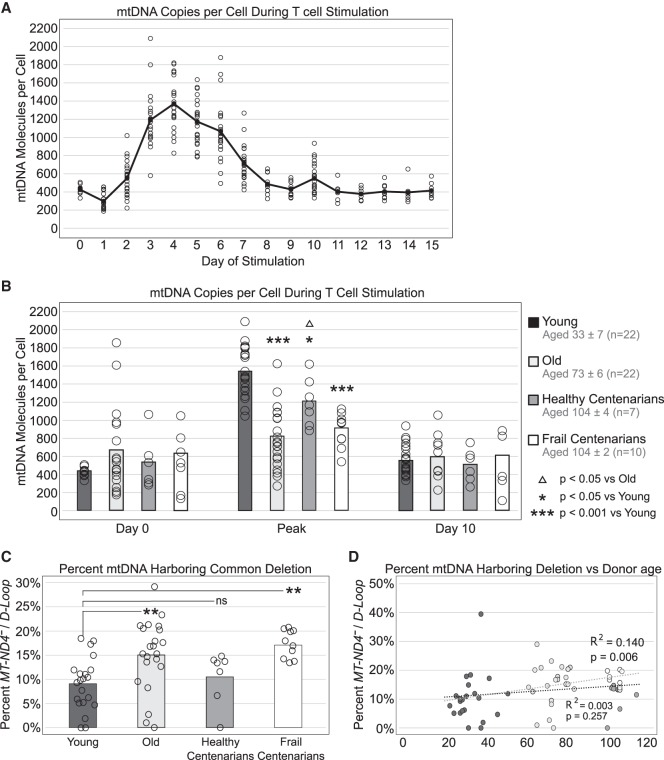
mtDNA in T cells shows stimulation- and age-dependent changes. (*A*) T cells from 22 healthy donors (aged 33 ± 7) were stimulated at day 0 by anti-CD3/CD28 beads and mtDNA per cell equivalent was measured over a 15-d period of stimulation. Open circles represent individual mtDNA per cell from each of the 22 individuals, while the line traces the average of all individuals at each day. (*B*) T cells from 22 healthy young (aged 33 ± 7), 22 healthy old (aged 73 ± 6), 7 healthy centenarian (aged 104 ± 4), and 10 frail centenarian (aged 104 ± 3) individuals were stimulated as described above. mtDNA was measured over the course of the stimulation, and the highest point that mtDNA reached for each individual was designated as the “peak” mtDNA of that individual's stimulation. Shown are averages and standard deviation of the peak mtDNA of each age group. Significance was determined by one-way ANOVA. (*C*) *MT-ND4* and *D-Loop* molecules were measured from T cells to show the prevalence of mutated mtDNA (carrying the 4977-bp common deletion) to total mtDNA. Significance was determined by ANOVA (Tukey's multiple comparisons). (*D*) The percentage of mtDNA carrying *MT-ND4* deletion was plotted versus age for all 61 study participants. Linear regressions were performed for young, old, and frail centenarians (gray line) and young, old, and healthy centenarians (black line).

Consistent with previous studies (Dimeloe et al. 2017; D'Souza et al. 2007), upon stimulation, mtDNA levels were transiently up-regulated, generally peaking 3–6 d after stimulation and then slowly declining (Fig. 4A; Supplemental Table S4). A small but significant dip in mtDNA per cell can be seen at day 1 (422 ± 44 vs. 298 ± 85 at days 0 and 1, respectively; *P* < 0.0001), possibly indicative of activation-induced cell death occurring in a subset of cells (Kabelitz et al. 1993). Unstimulated PBMCs (day 0) showed lower levels of mtDNA, in line with these cells being in a quiescent state, and no significant differences were detected between the study groups (Fig. 4B; Supplemental Table S4). Although previous studies have reported decreased mtDNA in blood during aging (Kim et al. 2013; Mengel-From et al. 2014; Wachsmuth et al. 2016), our study found no significant differences. While we measured mtDNA in resting PBMCs (mostly T cells) cultured in vitro, most previous studies employed DNA isolated from whole blood. In blood, there are free-circulating mtDNA molecules, granulocytes (not part of PBMCs), and both red blood cells and platelets (that contain mtDNA but no genomic DNA), suggesting that the ratio mtDNA/nDNA in whole blood could be different compared to PBMCs alone (Hurtado-Roca et al. 2016). Another possibility is that the employed culture conditions (cells were kept in culture for at least 24 h before stimulation) slightly altered the cell physiological mtDNA copy number of resting PBMCs.

However, during stimulation a noticeable impairment in mtDNA biogenesis can be seen with increased age, as measured by the mtDNA copy number peak of each individual over the course of the stimulation period (809 ± 332 vs. 1528 ± 272 in old vs. young, respectively; *P* < 10^−8^) (Fig. 4B; Supplemental Table S4). This suggests an age-related impairment in PBMCs’ ability to up-regulate mtDNA biogenesis in response to antigen-specific activation. However, we found that the mtDNA copy number peak was significantly higher in healthy centenarians as compared to old (aged 73 ± 6; 1198 ± 261 vs. 809 ± 332; *P* < 0.05) (Fig. 4B; Supplemental Table S4). Given the importance of this data, we further confirmed the mtDNA copy number in stimulated PBMCs from healthy and frail centenarians by repeating mtDNA quantitation using the other previously validated sets of primers amplifying the *MT-ND1* and *MT-TL1* regions (Supplemental Fig. 1A,B; Supplemental Table S5). We found a very significant correlation between mtDNA quantitation using *D-Loop* primers and mtDNA quantitation using both *MT-ND1* and *MT-TL1* primers (Supplemental Fig. 1E,F; Supplemental Table S5). Overall, these data support the idea that mtDNA biogenesis during immune cell stimulation may be a potential biomarker of healthy aging.

### ddMDM to quantify common mtDNA deletions: a pilot study in centenarians

Over the course of an individual's lifespan, mtDNA mutations and deletions accumulate within cells and tissues; in some instances, mutant mtDNA propagates at an even faster rate than its wild-type counterparts (Trifunovic et al. 2004; Lakshmanan et al. 2018). It has been suggested that such age-related increases in mtDNA heteroplasmy may contribute to the onset and progression of various diseases (Cortopassi et al. 1992; Chen et al. 2011). The most frequent mtDNA mutation is the “mtDNA common deletion,” a 4977-bp deletion occurring between two 13-bp direct repeats, resulting in the disruption of mitochondrial complexes I, IV, and V (Cortopassi and Arnheim 1990; Krishnan et al. 2007; Meissner et al. 2008). In the present study, we adapted ddMDM to multiplex *MT-ND4*, a gene lying within the deletion, and the *D-Loop*, a region present in all replication-competent mtDNA molecules, such that the proportion of mtDNA molecules carrying the 4977-bp common deletion can be calculated as 1 – *MT-ND4*/*D-Loop*. Primer and probe sequences are listed in Supplemental Material.

As expected, stimulated PBMCs from young adults displayed a significantly lower prevalence of the “mtDNA common deletion” as compared to both old (aged 73 ± 6) and frail centenarians (Fig. 4C; Supplemental Table S4). Further, when we performed a linear regression correlating the percentage of mtDNA molecules carrying the common deletion with age in young, old, and frail centenarians, we observed a significant age-related decrease in the percentage of intact (unmutated) mtDNA (R^2^= 0.140; *P* = 0.006) (Fig. 4D; Supplemental Table S4). In addition, we found no significant difference between young adults and healthy centenarians (Fig. 4C; Supplemental Table S4), and when we substituted healthy centenarians for frail centenarians and performed the same linear regression, we no longer observed a significant correlation (R^2^= 0.003; *P* = 0.257) (Fig. 4D; Supplemental Table S4). Together, these data can be interpreted to show that mtDNA deletions not only accumulate during aging but also that retaining more intact mtDNA appears to be a feature of healthy centenarians and healthy aging. Future investigations are required to explore whether the ability to sustain a higher pool of unmutated mtDNA is a consequence of good health and a relatively disease-free status or whether it promotes optimal health status and it is perhaps causal in some centenarians’ ability to maintain health into extreme age.

## Discussion

The growing number of epidemiological studies exploring the role of mtDNA in health and disease showcases the need for more sensitive and more efficient methods for mtDNA copy number quantification. qPCR is still a major method in the field of mtDNA biology and, as a result, mtDNA copy number measurements are often confounded by a lack of absolute quantification. The relative quantifications employed by most mtDNA investigations make comparisons between different studies difficult to interpret and present hurdles to understanding the roles of mtDNA copy number variation during aging and disease onset and progression. This is at least in part due to the following: (1) the differing PCR amplification efficiency between the mtDNA target and housekeeping genes, leading to altered mtDNA/nDNA ratios (Chen et al. 2007; Regier and Frey 2010); and (2) the manner in which DNA is isolated (Andreu et al. 2009; Malik et al. 2011). For example, DNA purified by the manual phenol method can give randomly false high mtDNA copy number values, while other column-based methods seem to be less variable (Andreu et al. 2009). However, since mtDNA is relatively small and exhibits different conformational structures (e.g., supercoiled, relaxed, or linear), it can be lost more easily than genomic DNA during DNA isolation, and different methods lead to different mtDNA/nDNA ratios (Chen et al. 2007).

By developing ddMDM, we were able to directly address these key limitations. In particular, the direct use of cell lysates foregoes the need of DNA purification and the complications described above, while mtDNA quantitation based on cell equivalents per the reaction eliminates the risk associated with differing PCR efficiencies between mtDNA and nuclear reference genes. This also has a critical upside in preventing biased results due to copy number variants of nuclear reference genes, which occur frequently in cancer. Results are presented as *D-Loop* molecules per cell equivalent, corresponding to number of mtDNA molecules per cell. ddMDM further provides the added benefit of reduced costs and workflow time and increased sensitivity to detect subtle alterations in mtDNA levels. This could be particularly important for studies aimed at investigating disease etiology and pathogenesis. For example, with this improved method, we were able to document, in an in vitro model of human colorectal cancer, that upon cancer initiation and early progression there is a slight but significant progressive decrease in mtDNA cellular content that correlates with phenotypic changes (Fig. 3C; Supplemental Table S3). This strengthens the hypothesis that metabolic reprograming is one of the key hallmarks during cancer progression (Ward and Thompson 2012) and that lower levels of mtDNA in certain cancers may reflect a more stem-like metabolic program (Lee et al. 2015; Menendez 2015). In addition, ddMDM allows for mtDNA quantifications in single cells, which was not possible using the other methods and may be important for future investigations of mtDNA and metabolic control at the single-cell level to better understand the complex heterogeneity and dynamic landscape predicated upon both cell autonomous and microenvironment features.

Our method also can be useful for epidemiologic studies which are often characterized by minor variations in mtDNA levels that are difficult to detect and often produce conflicting results across different investigations. For example, we observed a mild gradual age-related reduction in mtDNA copy number in stimulated PBMCs from a well-characterized human population aged 23–113. However, healthy centenarians appeared to retain significantly more mtDNA molecules and less major mtDNA deletions (Fig. 4B–D; Supplemental Table S4). We have recently identified and validated a number of genes involved in promoting immune cell stimulation (e.g., *CD28*) and anti-inflammatory pathways (e.g., *IL10*) whose expressions were significantly up-regulated in healthy centenarians (Tedone et al. 2019) and thus positively correlate with their increased mtDNA levels (Fig. 4B; Supplemental Table S4). We also identified a number of genes involved in detrimental cellular processes such as apoptosis (e.g., *BAX*, *PDCD1*) and chronic inflammation (e.g., *TNF*, *IL1A*, *NLRP3*) that were significantly down-regulated in healthy centenarians (Tedone et al. 2019) and negatively correlate with mtDNA levels (Fig. 4B; Supplemental Table S4). Overall, this suggests that retaining more mtDNA copies (and with more efficient maintenance) during immune cell stimulation and proliferation is a hallmark of healthy centenarians and going forward, mtDNA quantitation could be employed as a potential biomarker of healthy aging and as a monitoring biomarker of mitochondrial dysfunction.

There is an increasing need to establish clinical reference ranges of mtDNA copy number in blood to be used in clinical diagnosis and monitoring of mitochondrial diseases as well as other conditions. In this regard, new methods to directly estimate mtDNA copy number from whole-genome sequencing data have been recently developed (Ding et al. 2015; Cohen et al. 2016; Qian et al. 2017). These methods are based on the rationale that sequencing coverage should be proportional to the underlying DNA copy number for autosomal and mitochondrial DNA. Although mtDNA quantification through whole-genome sequencing data analysis requires complex computation and considerably higher costs, similarly to our method it provides absolute (not relative) measurement of mtDNA copy number (Ding et al. 2015; Cohen et al. 2016; Qian et al. 2017). These studies showed mtDNA copy number in resting blood immune cells to range between 50 and around 800 (Ding et al. 2015; Cohen et al. 2016), and this is in line with our quantitations by using ddMDM (Fig. 4B, [day 0]; Supplemental Table S4).

In summary, we propose ddMDM as a novel high-throughput and rapid (3 h) method that provides highly quantitative and sensitive mtDNA measurements. ddMDM may be useful for epidemiologic studies, clinical monitoring of mitochondrial diseases, and for much needed studies aimed at better understanding the contribution that mtDNA plays in aging and disease, not only for its role in metabolism but also as a signaling molecule. In fact, even though the basis of any mechanism regulating mtDNA copy number remains a poorly understood phenomenon (Gammage and Frezza 2019), cellular mitochondrial content is believed to modulate global transcription (Guantes et al. 2015), but this is an area of interest that remains mostly unexplored.

## Methods

### Cell culture

Human PBMCs were obtained from 61 donors in accordance with the Institutional Review Board (IRB)-approved protocol (certification number STU 042014-016) (Tedone et al. 2019). Subjects affected by cancer, infections, or autoimmune diseases or on immunosuppressive treatment at the time of enrollment were excluded from the study. The participant's age at time of enrollment was defined by birth certificates or identity documents. A trained multidisciplinary staff collected from the enrolled volunteers information regarding their health status together with past and current disease history. Venous blood samples were drawn from the participants under fasting conditions at the same time in the morning. The study protocol was approved by the Ethical Committee of Saint Orsola - Malpighi University Hospital (Bologna, Italy), and written informed consent was obtained from all subjects in accordance with the International Declaration of Helsinki.

PBMCs were isolated by a density gradient centrifugation procedure as previously described (Tedone et al. 2015) and were then cryopreserved at −140°C pending analysis. Cells were thawed 24 h prior to stimulation and cultured in RPMI + GlutaMAX-I with 10% fetal bovine serum, 1% penicillin, streptomycin, and Amphotericin B (Gemini Bio-Products). After 24 h, the cell suspensions were transferred into a new flask to remove monocytes that tend to adhere to the plastic and can phagocytose Dynabeads used for T cell stimulation. PBMCs were stimulated by adding Dynabeads Human T-Activator CD3/CD28 (Life Technologies) in a 1:1 cell-to-bead ratio. After 72 h of stimulation, Dynabeads were removed using a magnet and cells were cultured for up to 15 d after stimulation. The percentage of live cells was determined every day by trypan blue exclusion using a TC20 Automated Cell Counter (Bio-Rad). Cell density was maintained at 1.0 × 10^6^ cells/mL.

Jurkat (T leukemia) cells were cultured in RPMI + GlutaMAX-I with 10% fetal bovine serum, 1% penicillin, streptomycin, and Amphotericin B (Gemini Bio-Products) and maintained at 1.0 × 10^6^ cells/mL. H1299 (nonsmall cell lung adenocarcinoma), 293T (human embryonic kidney), RPE (retinal pigment epithelial), and various populations of normal human BJ (foreskin fibroblasts) cells were cultured in ambient oxygen, 5% CO_2_, and maintained in a 4:1 ratio of DMEM (Dulbecco's Modified Eagle's Medium, GE Healthcare) to Medium 199 containing 10% calf serum (HyClone). For quantification of mtDNA copy number at different phases of the cell cycle (Supplemental Fig. 1D; Supplemental Table S5), H1299 cells were blocked in G1/S phase by treating with 2.5 mM thymidine for 16 h. Cells were then washed four times with PBS and cultured for 8 h. Finally, cells were blocked again with 2.5 mM thymidine for 16 h, washed four times with PBS and then cultured as described above.

Primary HBECs were grown in 2% oxygen using a 1:1 mixture of bronchial epithelial basal medium (BEBM, Lonza) and DMEM and supplemented with SingleQuot (Lonza) and 1% PSA. Primary dermal foreskin fibroblasts (early passage BJ fibroblasts) were isolated and cultured as previously described (Ramirez et al. 2003).

The HCEC cancer progression series lines (1CT, 1CTP, 1CTR, 1CTRPA, 1CTRPM) were grown as described above. Initially, diploid HCECs lines were established by *TERT* (T) and *CDK4* (C) immortalization. Then, additional lines bearing combinations of commonly found mutations during colorectal cancer initiation and early development were established. (A: *APC* shRNA stable knockdown and truncated *APC* A1309 overexpression; M: *MYC* overexpression; P: *TP53* shRNA stable knockdown; R: mutant *KRAS*^*v12*^ overexpression) (Eskiocak et al. 2011; Zhang et al. 2015).

### Cell lysis procedure for mtDNA quantification

Pellets containing 1 × 10^5^ cells were collected and stored at −80°C pending analysis. Cell pellets were lysed on ice using 40 µL of NP-40 lysis buffer (10 mM Tris-HCl, pH 8.0, 1 mM MgCl_2_, 1 mM EDTA, 1% [vol/vol] NP-40, 0.25 mM sodium deoxycholate, 10% [vol/vol] glycerol, 150 mM NaCl, 5 mM β-mercaptoethanol, 0.1 mM AEBSF [4-(2-aminoethyl)benzenesulfonyl fluoride hydrochloride]) and vortexed every 15 min for a total of 45 min. Once lysed, 10 µL of lysate (containing 2.5 × 10^4^ cell equivalents) were added to 9 µL of TNES buffer (10 mM Tris pH 8.0, 100 mM NaCl, 10 mM EDTA, 1% SDS) and 1 µL of Proteinase K (20 mg/mL, Invitrogen). Proteinase K digestion was performed at 50°C for 15 min, followed by 100°C for 10 min, then cooled to 12°C. Following Proteinase K treatment, samples were diluted with ddH_2_O 1:250 to obtain the final concentration of 5 cell equivalents per µL. After dilution, samples were prepared for ddPCR. ddPCR is sensitive to oversaturation of template molecules, and optimal sample dilution will vary between cell types, dependent on mtDNA content per cell.

### ddPCR by using cell lysates (ddMDM)

Each 20-µL ddPCR reaction contained a final concentration of 1× EvaGreen ddPCR Supermix (Bio-Rad), 100 nM *D-Loop* forward, 100 nM *D-Loop* reverse primer, and 1 µL of sample (1–5 cell equivalents, depending on cell line). Samples were partitioned into droplets using a QX100 droplet generator (Bio-Rad) according to the manufacturer's instructions, and the emulsions (∼40 µL) were transferred to a 96-well PCR plate (twin-tec, Eppendorf) and sealed with foil (Thermo Fisher Scientific, AB0757). Following droplet formation, PCR was performed at 95°C for 5 min, 40 cycles of 95°C, 54°C, and 72°C for 30 sec each, then held at 12°C. The ramp rate between all steps was 2.5°C/sec. After PCR, fluorescence was measured using a QX100 droplet reader (Bio-Rad), detecting, on average, 15,000–20,000 droplets per sample. The threshold for positive droplets was determined by the software's analysis of droplet clustering across all samples and confirmed manually by comparison to a negative, cell-free control (Fig. 1F; Supplemental Table S1). The final output given by the software was a concentration of starting template molecules per µL and could be converted to molecules per 20-µL reaction (the original pre-emulsion volume containing 1–5 cell equivalents). Finally, mtDNA per reaction was normalized to obtain copies of mtDNA per cell equivalent (ddMDM) (Fig. 1G; Supplemental Table S1).

For quantifications of mtDNA, expressed as molecules of mtDNA per single-copy nuclear gene (*D-Loop*/*NCOA3* ratio), two reactions per sample were prepared: one containing 100 nM *D-Loop* primers, the other containing 100 nM *NCOA3* primers. After PCR, the number of mtDNA copies per diploid genome was calculated as previously described (Bai and Wong 2005).

### Total genomic DNA extraction

Extraction and purification of total genomic DNA was performed as previously described (D'Souza et al. 2007). Briefly, 2 × 10^5^ cells were pelleted and resuspended in 250 µL of SDS lysis buffer. Samples were briefly vortexed then boiled for 10 min. Samples were cooled to room temperature and treated with 2.5 µL of RNase A (10 mg/mL, Qiagen) at 37°C for 2 h. Next, 2.5 µL of Proteinase K (10 mg/mL) was added, and samples were incubated at 55°C overnight. The next day, samples were boiled for 10 min, followed by DNA precipitation by adding 0.1 volumes of 3 M sodium acetate and 2 volumes of 100% ethanol at −20°C overnight. Centrifugation was performed at 4°C at 1500*g* for 15 min. Pellets were washed once in 70% ethanol and resuspended in 50 µL ddH_2_0. DNA concentration of samples was measured by a Qubit High Sensitivity DNA kit (Thermo Fisher Scientific).

### ddPCR by using purified genomic DNA

mtDNA measurements from genomic DNA by using ddPCR were performed as described (Wachsmuth et al. 2016). Briefly, total genomic DNA was isolated and ddPCR was performed as described above. Absolute mtDNA copies per ddPCR reaction were normalized to the single-copy nuclear gene *NCOA3*. In order to generate a dilution curve for normalized results, mtDNA was measured in serial dilutions of total genomic DNA and then normalized to the nDNA measured from 0.1 ng of the same DNA.

### qPCR

All qPCR reactions were performed as previously described (Bai and Wong 2005) and consisted of 1 µL of sample (1 ng total DNA), 1× Ssofast EvaGreen Supermix (Bio-Rad), 500 nM forward, and 500 nM reverse primer to a final volume of 20 µL. qPCR was performed using a LightCycler 480 (Roche) at 95°C for 5 min, 40 cycles of 95°C for 15 sec and 60°C for 60 sec, followed by melting curve analysis. Samples measured in qPCR represent the average of three technical replicates, unless otherwise indicated. In order to convert threshold cycles to absolute molecules, standard curves of known amounts of purified *D-Loop* and *NCOA3* amplicons were run concurrently with qPCR samples. Exponential regressions created from these standard curves were then used to calculate mtDNA and nDNA per ng of DNA employed. Dilution curves of normalized results were calculated as described above.

### Preparation of purified amplicons

One nuclear and three mitochondrial primer pairs were chosen, targeting *NCOA3* (Bai and Wong 2005), the *D-Loop* region (Bai and Wong 2005), *MT-ND1* (Kim et al. 2013), and *MT-TL1* (Bai and Wong 2005). These primers (sequences listed in Supplemental Material) were confirmed for specificity by both agarose gel and by qPCR melting curve analysis. PCR amplicons of these four primer pairs were then purified from 1% agarose gels using a Qiagen Gel Extraction kit, and concentrations were measured with a Qubit High Sensitivity DNA kit (Thermo Fisher Scientific). Each amplicon was then diluted to 1 × 10^9^ molecules per µL, aliquoted, and stored at −20°C.

### Single-cell mtDNA quantification

Forty-eight hours prior to collection, cells were cultured at low density in order to avoid potential contact-induced inhibition at the single-cell level. After trypsinization, PBS was added to adjust the cell density to ∼1 × 10^6^ cells/mL; 1 × 10^5^ cell pellets were collected to be simultaneously run alongside single-cell samples as pooled control samples. Single-cell isolation was performed as described previously (Huang et al. 2017). In brief, cells were diluted in 1× PBS + 0.1% FBS to 2000 cells/mL, and 1-µL aliquots were placed on a glass slide. Drops containing exactly one single cell were identified under the microscope, and then each visually confirmed 1-µL aliquot was transferred to a PCR tube containing 0.05 µL Proteinase K (25 mg/mL, 1.25 µg final), 0.45 µL TNES buffer, 1 µL NP-40 lysis buffer (see above), and 7.5 µL MS2 RNA in ddH_2_O (32 pg/µL, 240 pg final). MS2 RNA helped to prevent excessive quantities of DNA from sticking to tubes during single-cell reactions. The final 10-µL reaction was run in a thermocycler at 50°C for 30 min, 100°C for 10 min, then cooled to 12°C for lysis and Proteinase K treatment. Fifteen microliters of ddH_2_O were then added and the sample mixed thoroughly by pipetting; 7.5 µL of diluted sample (corresponding to 0.3 cell equivalents) was then used to perform ddPCR reactions as described above, with the exception of differing sample volumes. All single-cell ddMDM reactions shown were run in triplicate.

Alternatively, single cells can be isolated by FACS so that each cell will be automatically placed in a separate well of a 96-well PCR plate containing 0.05 µL Proteinase K (25 mg/mL, 1.25 µg final), 0.45 µL TNES buffer, 1 µL NP-40 lysis buffer (see above), and 8.5 µL MS2 RNA in ddH_2_O (28.2 pg/µL, 240 pg final). After this step, mtDNA quantification can be performed as described above. However, we noted that single-cell isolation via FACS often produced “false negative” (mtDNA copy number in single cells resulted to be zero). This likely occurred due to the sorter not aliquoting a cell in the well or the cell was not directly aliquoted into the lysis buffer at the bottom of the well (these results were excluded).

Therefore, we suggest employing the “operator performed” single-cell isolation as described above and by Huang et al. (2017).

### Statistical analysis

Statistical analyses were performed using Student's *t*-test (unpaired, two-tailed), ANOVA (Tukey's multiple comparisons), or linear regression where appropriate. Significance was denoted as follows: (*) *P* ≤ 0.05; (**) *P* ≤ 0.01; (***) *P* ≤ 0.001.

## Supplementary Material

Supplemental Material
