# Supplementary material for: Quantitative mitochondrial DNA copy number determination using droplet digital PCR with single-cell resolution
Source: Genome Res. 2019 Nov;29(11):1878–88. doi: 10.1101/gr.250480.119 (PMC6836731; doi:10.1101/gr.250480.119)
Supplement: Supplemental Material [file supp_29_11_1878__index.html]

Quantitative mitochondrial DNA copy number determination using droplet digital PCR with single-cell resolution — Supplemental Material 

# Quantitative mitochondrial DNA copy number determination using droplet digital PCR with single-cell resolution

## Supplemental Material

- Supplemental\_Fig\_S1.pdf.pdf
- Supplemental\_Fig\_S2.pdf.pdf
- Supplemental\_Material.pdf.pdf
- Supplemental\_Table\_S7.docx.docx
- Supplemental\_Table\_S5JDedit.xls
- Supplemental\_Table\_S3JDedit.xlsx
- Supplemental\_Table\_S4JDedit.xlsx
- Supplemental\_Table\_S1JDedit.xlsx
- Supplemental\_Table\_S2JDedit.xlsx
- Supplemental\_Table\_S6JDedit.xls
