# Supplementary material for: Quantitative mitochondrial DNA copy number determination using droplet digital PCR with single-cell resolution
Source: Genome Res. 2019 Nov;29(11):1878–88. doi: 10.1101/gr.250480.119 (PMC6836731; doi:10.1101/gr.250480.119)
Supplement: Supplemental Material [file supp_gr.250480.119_Supplemental_Fig_S1.pdf.pdf]

Supplemental Figure 1

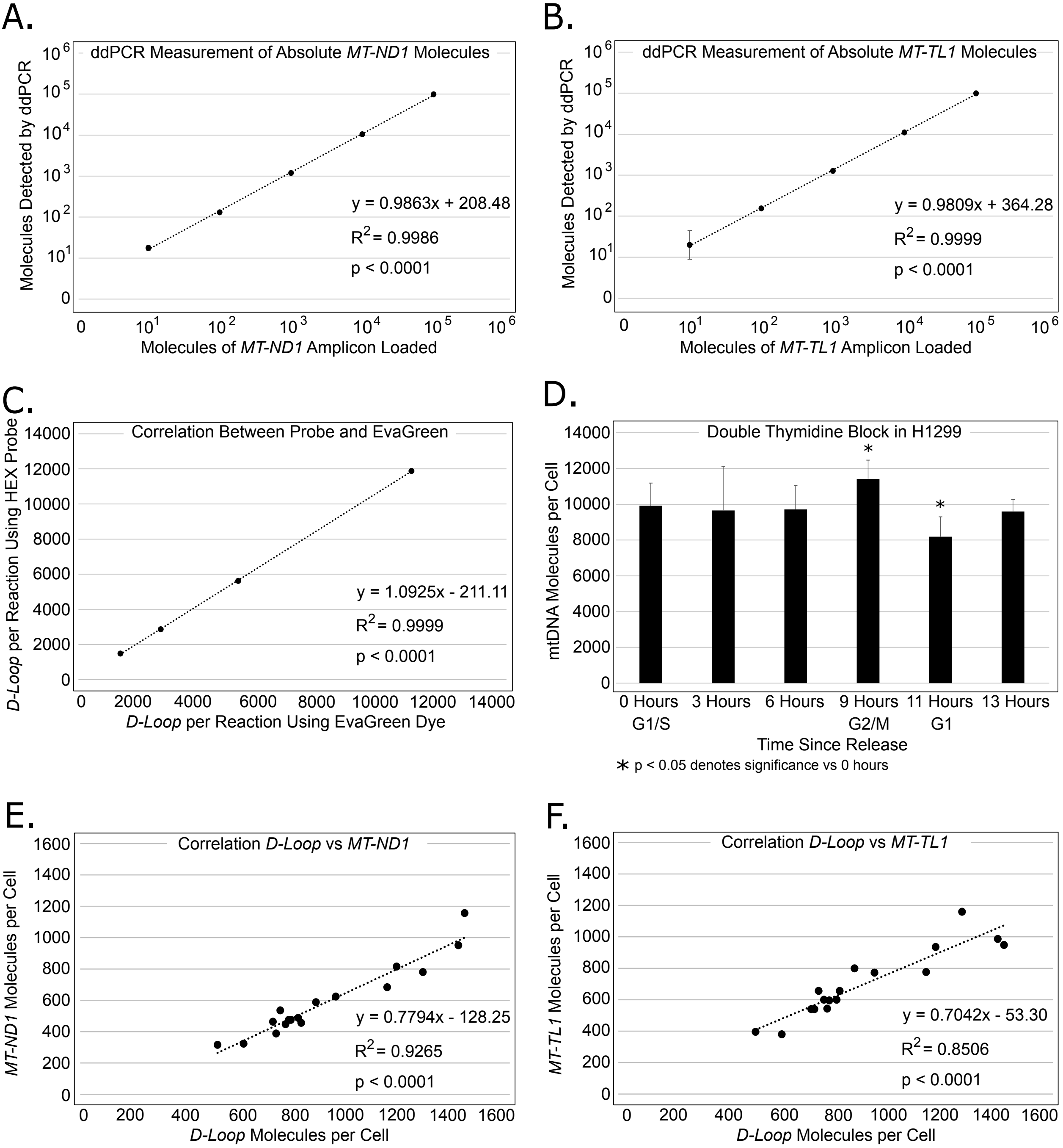

Supplemental Figure 1

Absolute quantification of additional mtDNA amplicons using ddPCR were measured in 10-fold dilution series. Axis are displayed in log10scale. Error bars show the standard deviation of four technical replicates. **A.***MT-ND1*, NADH Dehydrogenase Subunit 1. **B.***MT-TL1*, Mitochondrially-encoded tRNA Leucine 1 (UUA/G). **C.**Correlation between use of Taq-man probe and use of EvaGreen dye in the quantification of *D-Loop* molecules, performed in a 2-fold dilution series from stimulated T cells. **D.** mtDNA levels in H1299 cells at the indicated time points post-release from G1/S. Error bars represent the standard deviation of six biological replicates (two separate synchronizations run in triplicate). **E.** Correlation *D-Loop* vs *MT-ND1* in stimulated T cells from centenarians (peak). **F.** Correlation *D-Loop* vs *MT-TL1* in stimulated T cells from centenarians (peak).
