# Supplementary material for: Quantitative mitochondrial DNA copy number determination using droplet digital PCR with single-cell resolution
Source: Genome Res. 2019 Nov;29(11):1878–88. doi: 10.1101/gr.250480.119 (PMC6836731; doi:10.1101/gr.250480.119)
Supplement: Supplemental Material [file supp_gr.250480.119_Supplemental_Fig_S2.pdf.pdf]

Supplemental Figure 2

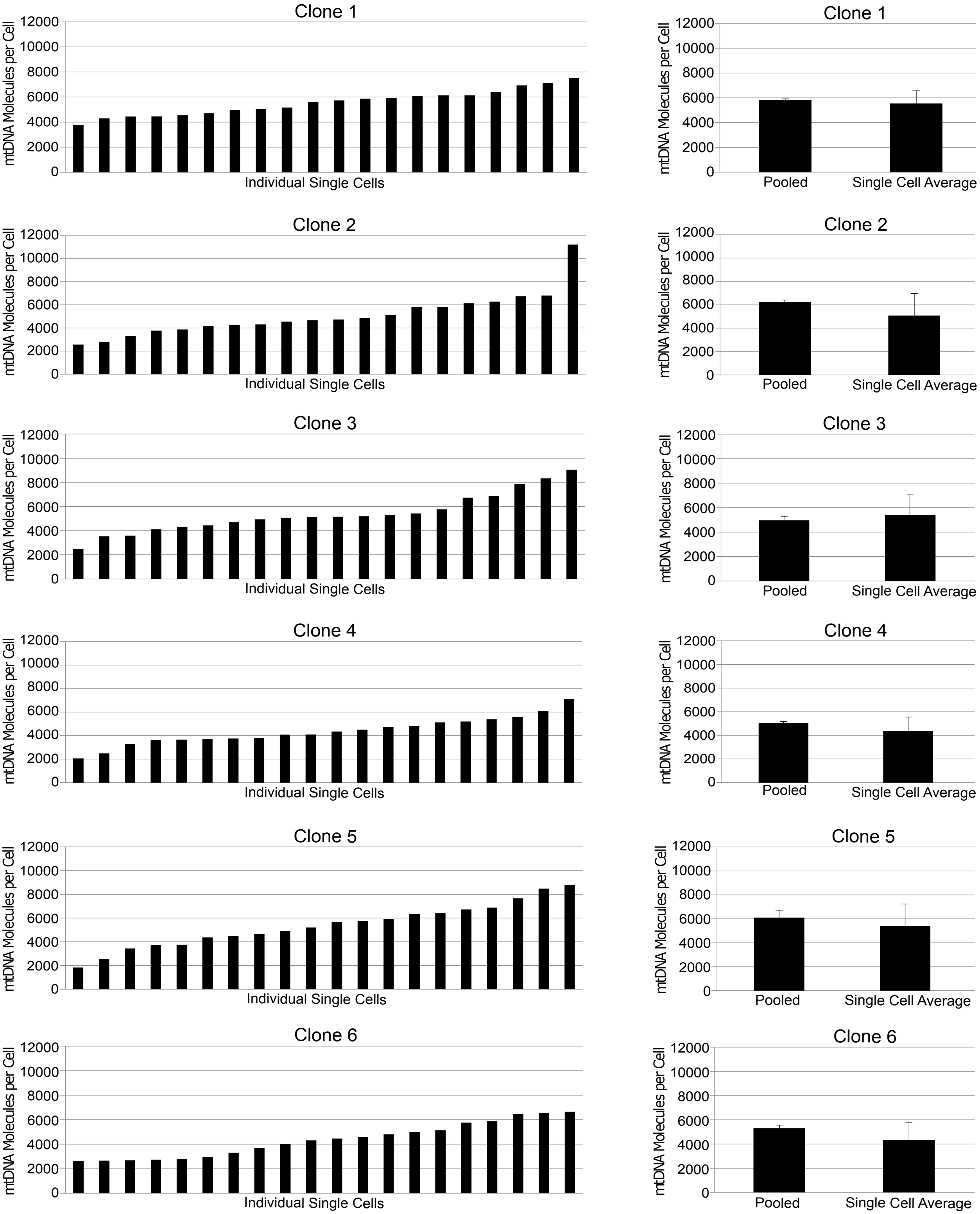

Supplemental Figure 2

ddMDM quantification of mtDNA copy number in H1299 single cells from different clones and comparisons between averages of H1299 pooled controls and averages of H1299 single cells.
