# Supplementary material for: Quantitative mitochondrial DNA copy number determination using droplet digital PCR with single-cell resolution
Source: Genome Res. 2019 Nov;29(11):1878–88. doi: 10.1101/gr.250480.119 (PMC6836731; doi:10.1101/gr.250480.119)
Supplement: Supplemental Material [file supp_gr.250480.119_Supplemental_Material.pdf.pdf]

Primer sequence

D-Loop:

F: CATCTGGTTCCTACTTCAGGG

R: TGAGTGGTTAATAGGGTGATAGA

Amplicon size: 104 bp

Region: 16498 - 32

Sequence used: https://www.mitomap.org/foswiki/bin/view/MITOMAP/HumanMitoSeq

MT-TL1:

F: CACCCAAGAACAGGGTTTGT

R: TGGCCATGGGTATGTTGTTA

Amplicon size: 108 bp

Region: 3212 - 3319

Sequence used: https://www.mitomap.org/foswiki/bin/view/MITOMAP/HumanMitoSeq

MT-ND1:

F: AACATACCCATGGCCAACCT

R: AGCGAAGGGTTGTAGTAGCCC

Amplicon size: 153 bp

Region: 3304 - 3456

Sequence used: https://www.mitomap.org/foswiki/bin/view/MITOMAP/HumanMitoSeq

NCOA3 (AIB1) (single copy nuclear gene)

F: GAGTTTCCTGGACAAATGAG

R: CATTGTTTCATATCTCTGGCG

Amplicon size: 134 bp

Region: 739 - 872

Sequence used: GI:2331249

ddMDM to quantify common mtDNA deletions:

D-Loop primers:

F: CATCTGGTTCCTACTTCAGGG

R: TGAGTGGTTAATAGGGTGATAGA

D-Loop Probe (HEX Probe): [HEX]-AGCCCACACGTTCCCCTTAAATAAGAC-[BHQ1]

MT-ND4 primers:

F: CCATTCTCCTCCTATCCCTCAAC

R: ACAATCTGATGTTTTGGTTAAACTATATTT

MT-ND4 Probe (FAM Probe): [6-FAM]-CCGACATCATTACCGGGTTTTCCTCTTG-[BHQ1]
