# Supplementary material for: Quantitative mitochondrial DNA copy number determination using droplet digital PCR with single-cell resolution
Source: Genome Res. 2019 Nov;29(11):1878–88. doi: 10.1101/gr.250480.119 (PMC6836731; doi:10.1101/gr.250480.119)
Supplement: Supplemental Material [file supp_gr.250480.119_Supplemental_Table_S7.docx.docx]

Supplemental Table 7

| Health status assessment | Healthier Centenarians  (n=7) | Frail Centenarians  (n=10) |
| --- | --- | --- |
| Age, years, mean ± S.D. | 105.6 ± 4.2 | 104.4 ± 2.0 |
| Gender, females | 5/7 | 7/10 |
| Smokers, % | 0 | 0 |
| Body Mass Index (BMI), mean ± S.D. | 24.1 ± 2.0 | 21.9 ± 2.7 |
| Disease count per individual, mean ± S.D. | 1.7 ± 0.5* | 4.8 ± 1.6 |

* p < 0.05

**Supplemental Table 7**

Health status assessment of centenarians. Disease count was determined by evaluating the presence of the following diseases: acute myocardial infraction, stroke, angina, hypertension, COPD, dementia, depression, diabetes, thyroid dysfunction, arthrosis, chronic liver diseases and chronic kidney diseases.
